# A Machine-Learning Approach to Developing a Predictive Signature Based on Transcriptome Profiling of Ground-Glass Opacities for Accurate Classification and Exploring the Immune Microenvironment of Early-Stage LUAD

**DOI:** 10.3389/fimmu.2022.872387

**Published:** 2022-05-26

**Authors:** Zhenyu Zhao, Wei Yin, Xiong Peng, Qidong Cai, Boxue He, Shuai Shi, Weilin Peng, Guangxu Tu, Yunping Li, Dateng Li, Yongguang Tao, Muyun Peng, Xiang Wang, Fenglei Yu

**Affiliations:** ^1^ Department of Thoracic Surgery, The Second Xiangya Hospital of Central South University, Changsha, China; ^2^ Hunan Key Laboratory of Early Diagnosis and Precise Treatment of Lung Cancer, The Second Xiangya Hospital of Central South University, Changsha, China; ^3^ Department of Ophthalmology, The Second Xiangya Hospital of Central South University, Changsha, China; ^4^ White Plains, NY, United States; ^5^ Key Laboratory of Carcinogenesis and Cancer Invasion, Ministry of Education, Department of Pathology, Xiangya Hospital, Central South University, Changsha, China; ^6^ National Health Council (NHC) Key Laboratory of Carcinogenesis (Central South University), Cancer Research Institute and School of Basic Medicine, Central South University, Changsha, China

**Keywords:** GGO (ground-glass opacity), LUAD, TCGA, GEO, prognosis

## Abstract

Screening for early-stage lung cancer with low-dose computed tomography is recommended for high-risk populations; consequently, the incidence of pure ground-glass opacity (pGGO) is increasing. Ground-glass opacity (GGO) is considered the appearance of early lung cancer, and there remains an unmet clinical need to understand the pathology of small GGO (<1 cm in diameter). The objective of this study was to use the transcriptome profiling of pGGO specimens <1 cm in diameter to construct a pGGO-related gene risk signature to predict the prognosis of early-stage lung adenocarcinoma (LUAD) and explore the immune microenvironment of GGO. pGGO-related differentially expressed genes (DEGs) were screened to identify prognostic marker genes with two machine learning algorithms. A 15-gene risk signature was constructed from the DEGs that were shared between the algorithms. Risk scores were calculated using the regression coefficients for the pGGO-related DEGs. Patients with Stage I/II LUAD or Stage IA LUAD and high-risk scores had a worse prognosis than patients with low-risk scores. The prognosis of high-risk patients with Stage IA LUAD was almost identical to that of patients with Stage II LUAD, suggesting that treatment strategies for patients with Stage II LUAD may be beneficial in high-risk patients with Stage IA LUAD. pGGO-related DEGs were mainly enriched in immune-related pathways. Patients with high-risk scores and high tumor mutation burden had a worse prognosis and may benefit from immunotherapy. A nomogram was constructed to facilitate the clinical application of the 15-gene risk signature. Receiver operating characteristic curves and decision curve analysis validated the predictive ability of the nomogram in patients with Stage I LUAD in the TCGA-LUAD cohort and GEO datasets.

## Introduction

Screening for early-stage lung cancer with low-dose computed tomography (LDCT) is recommended for high-risk populations; consequently, the incidence of pulmonary ground-glass opacity (GGO) is increasing (1). On CT, GGO appears as hazy opacities that do not obscure underlying pulmonary vessels or bronchial structures (2). GGO can manifest as benign or malignant lesions, including inflammation, preinvasive lesions, or adenocarcinomas (1). Typically, early lung adenocarcinomas (LUADs) *in situ* appear as pure ground-glass opacities (pGGOs), while advanced adenocarcinoma may appear as mixed ground-glass opacities (mGGOs) (3). pGGO and mGGO have significantly different prognoses, and solid LUADs are associated with shorter overall survival (OS) and recurrence-free survival compared to lesions with a GGO component (4, 5).

Guidelines on the management of GGO have been published (6–8); however, differentiating malignant and benign GGOs and clinical decision-making on the need for and timing of surgical resection are controversial (6, 9, 10). Persistent GGOs may represent premalignant conditions. Surgery involving wedge resection or segmentectomy, with or without regional lymph node dissection, is the most effective therapy for these patients (5, 11, 12). Most patients with GGO have satisfactory 5-year OS after appropriate therapy (4, 5); however, GGO may grow or demonstrate malignancy in approximately 20% of patients with pGGO and 40% of patients with mGGO (10, 13). A better understanding of the natural history of GGO, improved technology for diagnosis and follow-up, and establishing a precise size threshold for intervention may advance the management of patients with GGO (6, 13–17).

There is an unmet clinical need to understand the pathology of small GGO (<10 mm in diameter) (18–20), the factors associated with GGO growth and progression (21), and how evolving technology, including next-generation sequencing (NGS) combined with clinicopathological information, can facilitate a more accurate diagnosis of early-stage lung cancer (22). The objective of this study was to use the transcriptome profiling of pGGO specimens <1 cm in diameter to 1) construct a 15-gene risk signature to predict the prognosis of early-stage LUAD and 2) explore the immune microenvironment of GGO. Findings may inform a new classification strategy for early-stage lung cancer and improve diagnosis, follow-up, and treatment strategies.

## Methods

### Specimen Collection

All specimens were collected from patients undergoing surgery in the Second Xiangya Hospital of Central South University from May 2020 to May 2021. Specimens were stored at -80°C until analysis. Inclusion criteria were 1) pGGO < 1 cm in diameter detected with high-resolution CT (HRCT), 2) the patient underwent surgical resection and pathological analysis for clinical decision-making, and tumors were staged according to the American Joint Commission on Cancer (AJCC) 8th edition TNM staging system, and 3) postoperative pathological diagnosis confirmed LUAD. Finally, 30 paired samples of pGGO and adjacent normal tissue were sent to BGI Tech SOLUTIONS (Hongkong) for high-throughput transcriptome sequencing. The clinical characteristics of the patients with pGGO are summarized in **Table 1**.

**Table 1:** Clinical characteristics of patients.

Clinical characteristics of patients	
**Patients (n = 30)**	
NSCLC patients	30 (50.0%)
Non-cancer controls	30 (50.0%)
**Genders (n = 30)**	
Male	8 (26.7%)
Female	22 (73.3%)
**Age (n = 30)**	
≤ 60	25 (83.3%)
> 60	5 (16.7%)
**Sampling methods (n = 30)**	
Bronchoscopy	24 (80.0%)
Lobectomy	6 (20.0%)
**Smoking status (n = 30)**	
Smoker	10 (33.3%)
Non-smoker	20 (66.7%)
**TNM stage (n = 30)**	
I–II	30 (100%)
III–IV	0 (0%)
**pathological type**	
Adenocarcinoma in situ	6 (20.0%)
Minimally invasive adenocarcinoma	7 (23.3%)
Poorly-differentiated adenocarcinoma	4 (13.3%)
Moderately-differentiated adenocarcinoma	5 (16.7%)
Well-differentiated adenocarcinoma	8 (26.7%)
**GGO type**	
Single pure GGO	20 (66.7%)
Multiple pure GGO	10 (33.3%)

### Data Standardization and Differential Gene Expression Between pggo and Adjacent Normal Tissue

The high-throughput transcriptome sequencing dataset was normalized using the “edgeR” package in R (23). Differentially expressed genes (DEGs) between pGGO and adjacent normal tissue samples were obtained using the ‘‘Limma’’ package in R (|log FC|> 1, FDR P < 0.05) (24). pGGO-related DEGs were exhibited in a heatmap and volcano plot, which were generated by the “pheatmap, ggrepel, dplyr’’ package in R (25). Gene Ontology (GO) and Kyoto Encyclopedia of Genes and Genomes (KEGG) pathway enrichment analyses were conducted to identify the function of the DEGs.

### Non-Negative Matrix Factorization to Identify Molecular Subtypes

Next, we extracted the expression data of the pGGO-related DEGs in TCGA-LUAD stage I-II datasets. We also performed the non-negative matrix factorization (NMF) method to cluster the LUAD stage I–II patients. NMF is an unsupervised learning technique for dimension reduction that decomposes a large measurement matrix into two low-rank non-negative matrices (26). The cophenetic correlation coefficient, based on the consensus matrix and proposed by Brunet et al. (2004), is used to measure the stability of clusters (27). NMF can classify samples better than consensus clustering. mRNA expression profiles and the clinical data for 497 patients with LUAD (Stages I–II, n=347; Stages III–IV, n=121) were downloaded from The Cancer Genome Atlas (TCGA) (https://portal.gdc.cancer.gov/); follow-up information was available for each patient. The ID of the pGGO-related DEGs was used to extract expression data from the TCGA-LUAD cohort. The expression of the pGGO-related DEGs was verified in the TCGA-LUAD cohort. NMF was used to cluster patients with Stage I–II LUAD in the TCGA cohort. The clustering effect was evaluated with progression-free survival (PFS) and overall survival (OS).

### Identification of pGGO-Related DEGs and Differences in the Tumor Microenvironment Between the NMF Subgroups

Differences in pGGO-related DEGs between NMF subgroups were obtained using the “Limma” package in R. DEGs that were differently expressed between pGGOs and normal adjacent tissues, as well as differentially expressed between NMF subgroups, were identified. GO and KEGG pathway enrichment analyses were conducted to identify the function of the DEGs (28). The immune cell content of each NMF subgroup was analyzed using the “MCPcounter” package in R (29). HLA expression was compared between NMF subgroups. Based on a previous study, six immune subtypes were defined according to immune infiltrates. The relationship between the six immune subtypes and NMF subgroups was explored (30).

### Identification of pGGO-Related DEGs With Prognostic Value Using LASSO Cox Regression and Support Vector Machine—Recursive Feature Elimination

pGGO-related DEGs were screened with LASSO cox regression and support vector machine—recursive feature elimination (SVM-RFE) to identify prognostic marker genes (31). Feature selection for multiclass classification problems is challenging in machine learning. Existing multi-class gene selection algorithms are often not Pareto optimal. In this study, two machine learning algorithms, LASSO cox regression, and SVM-RFE, were used to achieve Pareto optimality (32, 33). LASSO cox regression is used for data dimensionality reduction and feature selection. The regression coefficient is penalized by L1, some coefficients are shrunk to zero, features with non-zero regression coefficients are selected, and 10-fold cross-validation is used to evaluate the prediction model (34). SVM-RFE is often used for gene selection. SVM-RFE ranks features from most important to least, and least important features are iteratively eliminated (35, 36).. Venn analysis was used to identify 15 pGGO-related DEGs that were shared between the LASSO cox regression and SVM-RFE machine learning algorithms.

### Validation of the pGGO-Related DEGs by the Quantitative Real-Time Reverse Transcription-Polymerase Chain Reaction

Quantitative real-time reverse transcription–polymerase chain reaction (qRT-PCR) was used to validate the expression of the pGGO-related DEGs with a prognostic value. RNA was extracted from 24 pGGO samples, and qRT-PCR was performed for 7 pGGO-related DEGs. Primer sequences were designed from Primer3web (https://primer3.ut.ee) (**Table 2**). qRT-PCR was performed with a SYBR Green super-mix reagent, and β-actin was the internal reference gene. The relative change in gene expression was calculated using the 2ΔΔCt method. Results are presented as the mean of 3 replicates.

**Table 2:** The primer sequence of the DEGs in risk signature.

Primer	Primer sequence (5'to 3')
PCP2-F	GAGAAGACGGAGGAAGGCTC
PCP2-R	CTCTGGCTCTTGGTGGTCTG
DKK1-F	CCATTGACAACTACCAGCCG
DKK1-R	TTTTGCAGTAATTCCCGGGG
KCNV1-F	CGGGAATTCTTGTCTTGGCC
KCNV1-R	CTCCATGATACTCCGGGCAT
FAIM2-F	AGCTTCCAGACCAAGTTCGA
FAIM2-R	TGTAAATACACCCGCTCCCA
FGF5-F	AGTGGTATGTGGCCCTGAAT
FGF5-R	TGGCTTGATAGGGCTAGGTG
NPAS1-F	CTTGTGAGAGCAGAGTCAGC
NPAS1-R	CTGCAGCCAACGGTAGTAAC
LINC00563-F	ATCTGGGATCATCTGGGTGG
LINC00563-R	CTTCCTGCATTCCTTCGCTC

### Establishment and Validation of the pGGO-Related DEG Signature

Multivariate Cox regression was used to calculate regression coefficients for the pGGO-related DEGs with prognostic values. A prognostic signature was constructed using the following formula: risk score = coefficient(gene1) * exp(gene1) +…+ coefficient (gene n) * exp (gene n). A deviation plot was constructed to show the expression profile of the 15 pGGO-related DEGs with prognostic values. Patients in the TCGA-LUAD cohort were stratified into a high-risk or low-risk group using the median risk score as a cut-off. The 15-gene risk signature was verified in the GSE50081 and GSE72094 datasets, which were downloaded from the Gene Expression Omnibus–NCBI (https://www.ncbi.nlm.nih.gov/geo/). Kaplan–Meier survival curves, decision curve analysis (DCA), risk plots, and time-dependent receiver operating characteristic (ROC) curves were used to investigate the predictive power of the prognostic signature in the TCGA-LUAD cohort and GEO datasets (37, 38). The relationship between clinicopathologic factors, immune scores, clusters, and risk scores was examined using Pearson’s chi-squared test (39).

### Clinical Application of the pGGO-Related DEG Signature

Univariate and multivariable Cox proportional hazard models were used to analyze independent associations between clinical outcomes. A nomogram based on clinicopathologic factors and risk scores was constructed. A calibration curve displayed the nomogram’s predictive power. Gene set enrichment analyses (GSEAs) in low-risk and high-risk groups were performed using the “clusterProfiler, enrichplot, DOSE, org.Hs.eg.db “package in R (40). Enrichment scores (ESs) that represent the degree to which a gene set is overrepresented at the top or bottom of a ranked list were calculated (https://www.gsea-msigdb.org/gsea/index.jsp). Tumor mutation burden (TMB) data were retrieved for the TCGA-LUAD cohort. The relationship between TMB and the 15-gene risk signature was evaluated using the “reshape2” package in R. The immunophenoscore (IPS) in patients with LUAD was downloaded from the Cancer Immunome Database (TCIA) (https://tcia.at/home) (41). The relationship between IPS and the 15-gene risk signature was evaluated with Pearson correlation analysis.

### Statistical Validation

All statistical analyses were conducted with R software (Version 4.0.1).

NMF algorithms were performed using the “NMF, survival” package (42). Kaplan–Meier survival curves were plotted using the “survminer” package. SVM-RFE was performed using the “e1071, kernlab, caret” package (43). The nomogram was constructed using the “rms” package. The predictive ability of the pGGO-related DEGs signature was evaluated using the “SurvivalROC” package. The deviation plot was constructed using the “ggpubr” package. Correlation analysis was performed using the Pearson method. Differences between subgroups were evaluated using the Wilcoxon rank-sum test. mRNA profiles from the GEO datasets were normalized using the “sva, Limma” package. All figures were plotted using the “ggplot” package. For the GO and KEGG analyses, a P-value <0.05 and FDR q-value <0.25 were considered statistically significant.

## Results

### Identification of DEGs Between pGGO and Adjacent Normal Tissue and NMF Clustering

**Figure 1:**
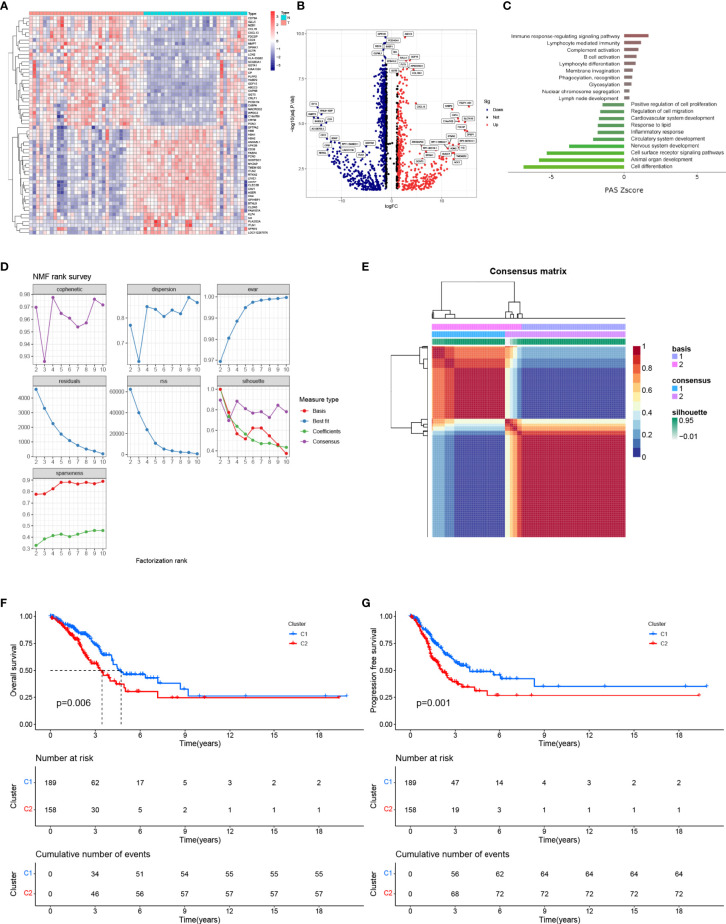
Identification of DEGs between pGGO and adjacent normal tissue and NMF clustering. **(A)** Heatmap of the DEGs (|log FC|> 1, FDR P < 0.05); **(B)** Volcano map of the DEGs (|log FC|> 1, FDR P < 0.05); **(C)** KEGG pathway enrichment analysis of the DEGs; **(D, E)** NMF clustering of patients with Stage I–II LUAD in the TCGA-LUAD cohort (**D** shows the NMF rank survey and E shows a heatmap of the consensus matrix; the best cluster number was chosen as the coexistence correlation coefficient K value = 2); **(F, G)** Kaplan–Meier survival curves for the NMF subgroups (**F** shows OS and **G** shows PFS). DEGs, differentially expressed genes; GGO, ground-glass opacity; KEGG: Kyoto Encyclopedia of Genes and Genomes; NMF, non-negative matrix factorization; LUAD, lung adenocarcinoma; PFS, progression-free survival; OS, overall survival.

A total of 1,734 DEGs (|log FC| > 2, P < 0.05) between pGGO and adjacent normal tissue samples were identified; of these, 648 DEGs were upregulated, and 1,086 DEGs were downregulated (**Figures 1A, B** and **Table S1**). GO and KEGG pathway enrichment analyses suggested that the DEGs were mainly involved in immune-related pathways, such as immune response-regulating signaling pathways and lymphocyte-meditated immunity pathways (**Figure 1C**). Next, we extract the mRNA expression data of the pGGO-related DEGs in TCGA-LUAD stage IA datasets and perform the NMF consensus cluster analysis. The best cluster number was chosen as the coexistence correlation coefficient K value = 2 (**Figure 1D**); therefore, patients with Stage I–II LUAD in the TCGA cohort (n = 347) were divided into two clusters (**Figure 1E** and **Table S2**). Patients in Cluster 1 had better PFS and OS compared to patients in Cluster 2 (**Figures 1F, G**).

### Identification of pGGO-Related DEGs and Differences in the Tumor Microenvironment Between the NMF Subgroups

**Figure 2:**
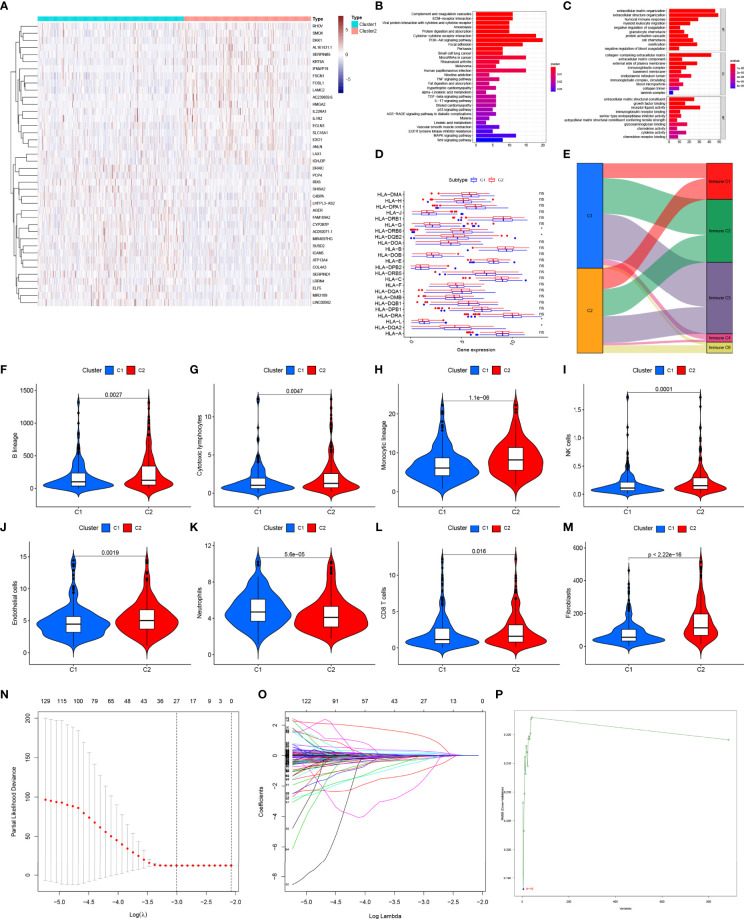
Identification of pGGO-related DEGs between Cluster 1 and Cluster 2 **(A)** Heatmap of the pGGO-related DEGs (|log FC|> 1, FDR P < 0.05); **(B, C)** KEGG **(B)** and GO **(C)** enrichment pathways analysis of the pGGO-related DEGs; **(D)** HLA gene expression data for Cluster 1 and Cluster 2; **(E)** Sankey diagram showing the relationship between the cluster subtype and six immune subtypes defined in a previous study; **(F–M)** Violin plots showing the expression of the immune cells in Cluster 1 and Cluster 2; **(N–P)** Selection of prognostic pGGO-DEGs in Cluster 1 and Cluster 2 using Lasso cox regression **(N, O)** and SVM-RFE (threshold value = 19) **(P)**. DEGs, differentially expressed genes; GGO, ground-glass opacity; KEGG, Kyoto Encyclopedia of Genes and Genomes; GO: Gene Ontology; NMF, non-negative matrix factorization; SVM-RFE: support vector machine—recursive feature elimination. ns represent non significant, * represent P≤ 0.05.

A total of 208 pGGO-related DEGs between Cluster 1 and Cluster 2 were identified; of these, 33 pGGO-related DEGs were upregulated, and 175 pGGO-related DEGs were downregulated (**Figure 2A** and **Table S3**). KEGG pathway enrichment analysis suggested that the pGGO-related DEGs were mainly enriched in immune-related pathways, including the cytokine−cytokine receptor interaction pathway and IL-17 signaling pathway (**Figure 2B**). GO function analysis suggested that the pGGO-related DEGs were mainly enriched in immune-related processes, including the humoral immune response process and immunoglobulin receptor-binding process (**Figure 2C**). HLA gene expression data showed that HLA-L, HLA-DQA2, HLA-DQB2, and HLA−DRB6 were highly expressed in Cluster 1 (**Figure 2D**). A Sankey diagram showed a relationship between the cluster subtype and six immune subtypes defined in a previous study (**Figure 2E** and **Table S4**). Violin plots suggested that Cluster 2 was characterized by a high expression of B-cell lineages, endothelial cells, cytotoxic lymphocytes, CD8 T cells, monocyte-lineage cells, fibroblasts, and NK cells; meanwhile, Cluster 1 was characterized by a high expression of neutrophils (**Figures 2F–M**).

Verification of pGGO-Specific DEGs Using Machine Learning Algorithms.

**Figure 3:**
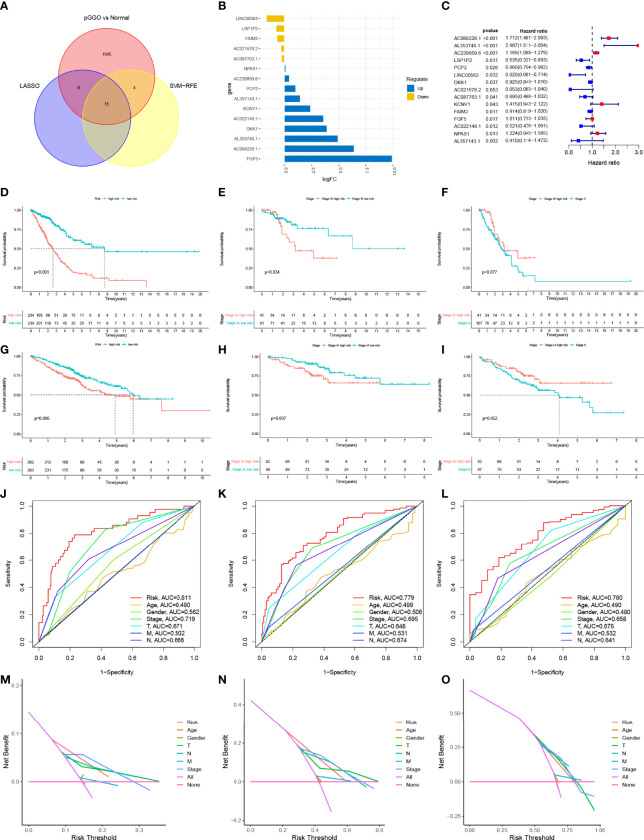
The construction and verification of the risk signature. **(A)** Venn analysis was used to identify 15 DEGs that were shared between the machine learning algorithms (Lasso cox regression and SVM-RFE); **(B)** Expression data of the 15 prognostic pGGO-related DEGs; **(C)** Forest plot of the 15 prognostic pGGO-related DEGs (red: risk factors; blue: protective factors); **(D–I)** Kaplan–Meier survival curves. The TCGA-LUAD cohort was stratified into a high-risk group and low-risk group. Patients in the high-risk group had a worse prognosis than the patients in the low-risk group in the overall TCGA-LUAD cohort **(D)** and patients with Stage IA LUAD **(E)**. There was no significant difference in OS between patients with Stage IA LUAD and high-risk scores and patients with Stage II LUAD **(F)**. Patients with LUAD from the GSE50081 and GSE72094 datasets were stratified into a high-risk group and low-risk group. Patients in the high-risk group had a worse prognosis than the patients in the low-risk group in the overall GEO-LUAD dataset **(G)** and patients with Stage IA LUAD **(H)**. There was no significant difference in OS between patients with Stage IA LUAD and high-risk scores and patients with Stage II LUAD **(I)**. **(J–L)** R Time-dependent ROC curve analysis at 1 **(J)**, 3 **(K)**, and 5 years **(L)** verified the predictive performance of the 15-gene risk signature in the TCGA-LUAD cohort. **(M–O)** DCA at 1 **(M)**, 3 **(N)**, and 5 years **(O)** verified the predictive performance of the 15-gene risk signature in the TCGA-LUAD cohort. DEGs, differentially expressed genes; GGO, ground-glass opacity; ROC, receiver operating characteristic; LUAD, lung adenocarcinoma; SVM-RFE, support vector machine—recursive feature elimination; DCA, decision curve analysis.

Lasso cox regression and SVM-RFE identified the 23 (**Figures 2N, O** and **Table S5**) and 19 (**Figure 2P** and **Table S5**) most representative prognostic pGGO-related DEGs, respectively, from among the 208 pGGO-related DEGs between Cluster 1 and Cluster 2. Venn analysis was used to identify 15 DEGs that were shared between the machine learning algorithms (**Figure 3A**). These included *DKK1, NPAS1, AL357143.1, KCNV1, AC068228.1, AC239859.6*, and *FGF5* which were highly expressed in pGGO samples, and *AC087763.1, PCP2, FAIM2, AL357143.1, AC022148.1, AC021678.2, LSP1P2*, and *LINC00563* that were highly expressed in adjacent normal tissue samples (**Figure 3B**). This expression profile was validated by qRT-PCR (**Figure S1**). Regression coefficients were used to identify a 15-gene risk signature. A forest plot showed that *FGF5*, *AC239859.6*, *NPAS1*, *KCNV1*, *AC068228.1*, and *AL353746.1* were risk factors in LUAD; while *DKK1*, *LINC00563*, *AC021678.2*, *AL357143.1*, *AC022148.1*, *LSP1P2*, *AC087763.1*, *PCP2*, and *FAIM2* were protective factors in LUAD (**Figure 3C**).

The TCGA-LUAD cohort was stratified into a high-risk group and low-risk group (n=234) based on their median risk score (**Table S6**). Kaplan–Meier survival analysis showed that patients in the high-risk group had worse OS than patients in the low-risk group (P<0.001) (**Figure 3D**). The 15-gene risk signature was predictive of patients with Stage IA LUAD in the TCGA-LUAD cohort. This was expected as pGGO is recognized as a component of TNM Stage IA LUAD according to the AJCC 8th edition TNM staging system. Patients with Stage IA LUAD and high-risk scores in the TCGA-LUAD cohort had worse OS than patients with Stage IA LUAD and low-risk scores (P<0.001) (**Figure 3E**). There was no significant difference in OS between patients with Stage IA LUAD and high-risk scores and patients with Stage II LUAD (**Figure 3F**). To confirm the feasibility of the 15-gene risk signature, information for patients with LUAD from the GSE50081 and GSE72094 datasets was downloaded, combined, and stratified into a high-risk group (n=263) and low-risk group (n=262) based on the median risk score from the TCGA-LUAD cohort (**Table S7**). All patients, and patients with Stage IA LUAD, in the high-risk group had worse OS than patients in the low-risk group (**Figures 3G, H**); there was no significant difference in OS between patients with Stage IA LUAD and high-risk scores and patients with Stage II LUAD (**Figure 3I**). Time-dependent ROC curve analysis at 1, 3, and 5 years showed that the 15-gene risk signature had better predictive performance than other clinical traits in the TCGA-LUAD cohort (1 year: AUC=0.811; 3 years: AUC=0.779; 5 years: AUC=0.780; **Figures 3J–L**). DCA showed that the 15-gene risk signature had more clinical benefits than other clinical traits (**Figures 3M–O**).

### Clinical Application of the 15-Gene Risk Signature

**Figure 4:**
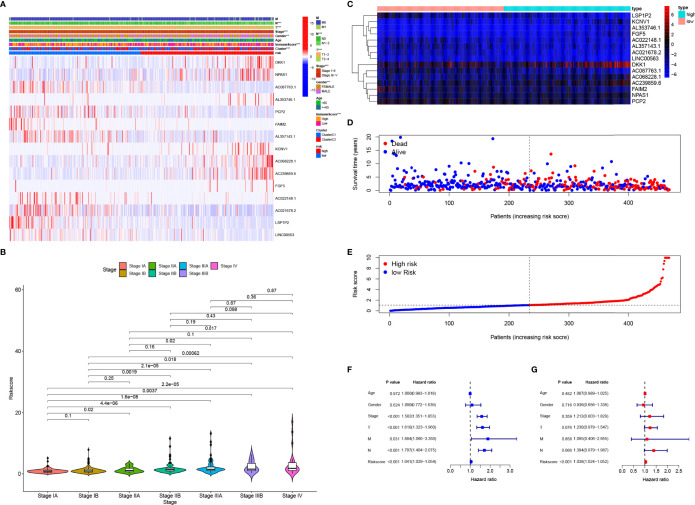
Clinical application of the 15-gene risk signature. **(A)** Heatmap showing that the TNM stage, immune scores, NMF subgroup, gender, T stage, and N stage were significantly associated with risk scores, and the expression levels of the 15 DEGs were different between the high- and low-risk groups. **(B)** Violin plots showing the relationship between the risk score and TNM stage. **(C–E)** Heatmap of the mRNA expression of the risk signature **(C)** and risk curves in the TCGA-LUAD cohort **(D, E)**. **(F, G)**, Univariate **(F)** and multivariate **(G)** Cox regression analyses suggested that the 15-gene risk signature was the independent prognostic factor in the TCGA-LUAD cohort. ROC, receiver operating characteristic curve; LUAD, lung adenocarcinoma; DCA, decision curve analysis. * represent P≤ 0.05, ** represent P≤ 0.01, *** represent P≤ 0.001, and **** represent P≤ 0.0001.

A heatmap showed that the TNM stage, immune scores, NMF subgroup, gender, T stage, and N stage were significantly associated with risk scores in TCGA datasets (**Figure 4A**, P<0.05). Most patients with high-risk scores were men (**Figure 4A**, P<0.001) and had higher-grade tumors (**Figure 4B**). Among the 15 pGGO-related DEGs in the gene risk signature, *DKK1, NPAS1, AL357143.1, KCNV1, AC068228.1, AC239859.6*, and *FGF5* were highly expressed in the high-risk group, and *AC087763.1, PCP2, FAIM2, AL357143.1, AC022148.1, AC021678.2, LSP1P2*, and *LINC00563* were highly expressed in the low-risk group (**Figure 4C**). Risk curves based on a per-sample risk score also validated the predictive power of the 15-gene risk signature (**Figures 4D, E**). Univariate and multivariate COX regression analyses showed that the 15-gene risk signature is an independent prognostic factor in patients with Stage I–II LUAD (**Figures 4F, G**: univariate Cox regression analyses: P<0.001, HR = 1.041; 95% CI: 1.029–1.054; multivariate Cox regression analyses: P<0.001, HR = 1.038; 95% CI: 1.024–1.052).

**Figure 5:**
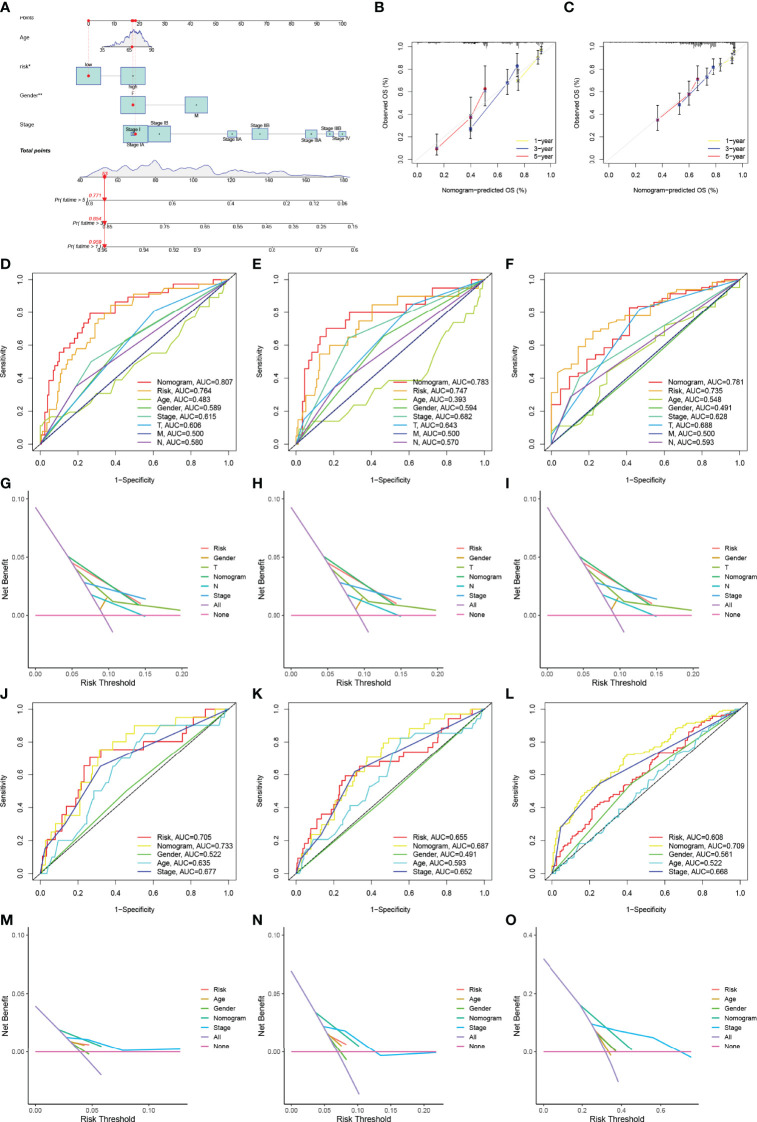
Construction and verification of the nomogram. **(A)** The nomogram was constructed using the TNM stage, risk signature, T stage, N stage, and gender. **(B, C)** Calibration curves verifying the performance of the nomogram at 1, 3, and 5 years in the TCGA-LUAD cohort **(B)** and GEO datasets **(C)**. **(D–F)** ROC curve analysis verifying the performance of the nomogram at 1 **(D)**, 3 **(E)**, and 5 years **(F)** in patients with Stage I LUAD in the TCGA cohort. **(D–F)** DCA verifying the performance of the nomogram at 1 **(G)**, 3 **(H)**, and 5 years **(I)** in patients with Stage I LUAD in the TCGA cohort; **(J–L)** ROC curve analysis verifying the performance of the nomogram at 1 **(J)**, 3 **(K)**, and 5years **(L)** in patients with Stage I LUAD in the GEO datasets. **(M–O)** DCA verifying the performance of the nomogram at 1 **(M)**, 3 **(N)**, and 5 years **(O)** in patients with Stage I LUAD in the GEO datasets. ROC, receiver operating characteristic curve; LUAD, lung adenocarcinoma; DCA, decision curve analysis. * represent P≤ 0.05, ** represent P≤ 0.01, *** represent P≤ 0.001, and **** represent P≤ 0.0001.

A nomogram incorporating clinical factors and the 15-gene risk signature was constructed for visualization and convenient clinical application (**Figure 5A**). Calibration curves validated the ability of the 15-gene risk signature to predict OS in the TCGA-LUAD cohort and LUAD dataset obtained from the GEO (**Figures 5B, C**). The ROC curve and DCA analysis validated that the nomogram had better predictive performance than the 15-gene risk signature alone at 1, 3, and 5 years in patients with Stage I LUAD from the TCGA-LUAD cohort (**Figures 5D–I**) and GEO (**Figures 5J–O**).

### The Relationship Between the 15-Gene Risk Signature, TMB, and the IPS

**Figure 6:**
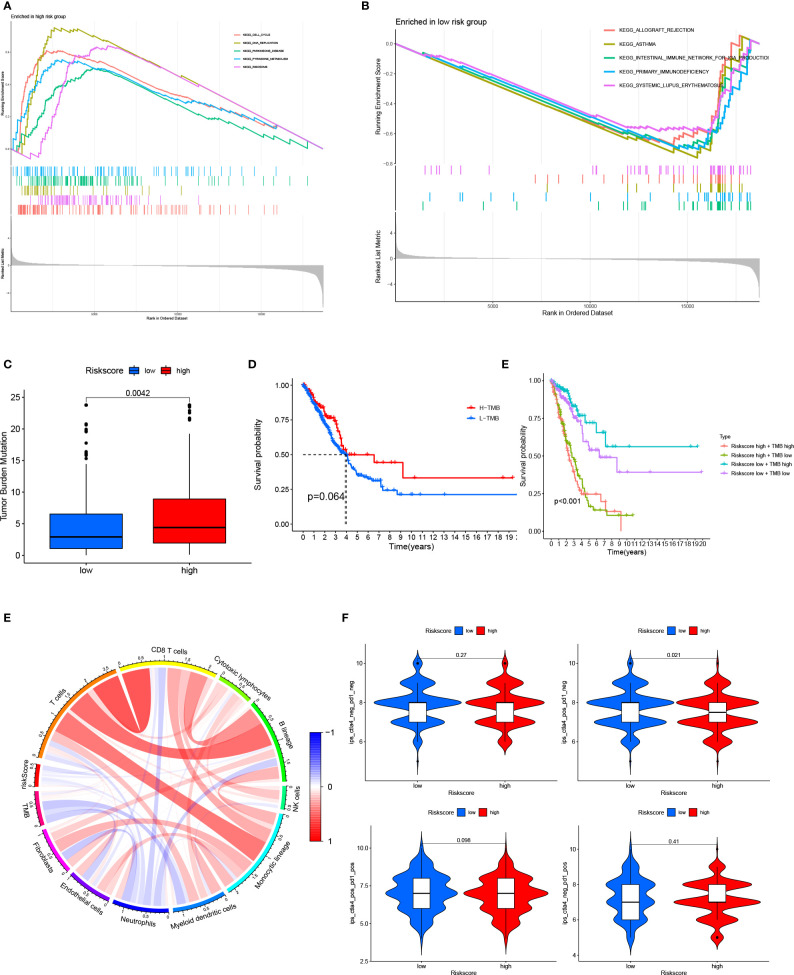
Relationship between the 15-gene risk signature, TMB, and the IPS **(A, B)** GSEA for patients in the high- and low-risk groups; **(C)** TMB in patients in the high- and low-risk groups; **(D)** Kaplan–Meier survival curves showed no significant difference in OS between patients in the high-TMB group and low-TMB group; **(E)** Kaplan–Meier survival curves combining the risk score and TMB; **(F)** Relationship between the 15-gene risk signature, TMB, and immune cells; **(G)**, Relationship between the 15-gene risk signature and the IPS. ROC, receiver operating characteristic; LUAD, lung adenocarcinoma; IPS, immunophenoscore; TMB, tumor mutation burden; OS, overall survival.

In the TCGA cohort, the GSEA suggested that the cell cycle, DNA replication, Parkinson’s disease, pyrimidine metabolism, and ribosome pathways were activated in patients with high-risk scores; meanwhile, allograft rejection, asthma, the intestinal immune network for IgA production, primary immunodeficiency, and systemic lupus erythematosus pathways were activated in patients with low-risk scores (**Figures 6A, B**). High-risk patients had higher TMB than low-risk patients (**Figure 6C** and **Table S8**). Patients were stratified into a high-TMB group and a low-TMB group based on the median TMB value. There was no significant difference in OS between patients in the high-TMB group and low-TMB group; however, patients with a high-risk score and a high TMB had the worse OS (**Figure 6D**). Patients with a low-risk score and high TMB had the best OS (**Figure 6E**). Patients with high-risk scores always had low levels of immune infiltration (**Figure 6F**). Patients with a low-risk score and tumors that were CTLA4 positive and PD1 negative had a high IPS, suggesting that these patients may benefit from immunotherapy (**Figure 6G**, **Table S9**).

## Discussion

The understanding of the pathogenesis of early-stage LUAD has increased with the advent of high-throughput transcriptome sequencing technology; however, the knowledge of the etiology and natural progression of pGGO is limited, especially for pGGO <1 cm in diameter (44). In this study, we identified DEGs between pGGO <1 cm in diameter and the adjacent normal tissue. Functional analysis of the DEGs suggested that the immune microenvironment plays an important role in LUAD tumorigenesis. NMF identified the 2 subgroups (Cluster 1 and Cluster 2) of patients with Stage I–II LUAD in the TCGA-LUAD cohort. The pGGO-specific DEGs between Cluster 1 and Cluster 2 mainly participated in immune-related pathways. Immune infiltrates in Cluster 2 were characterized by a high expression of B-cell lineages, endothelial cells, cytotoxic lymphocytes, CD8 T cells, monocyte-lineage cells, fibroblasts, and NK cells, while Cluster 1 was characterized by a high expression of neutrophils. Survival analysis showed that patients in Cluster 1 had a better prognosis than patients in Cluster 2. Taken together, these data suggest that a higher level of immune infiltrates may indicate a poor prognosis in patients with Stage I–II LUAD.

pGGO-related DEGs were screened to identify prognostic marker genes with two machine learning algorithms. A 15-gene risk signature was constructed from the DEGs that were shared between the algorithms. Risk scores were calculated using the regression coefficients for those pGGO-related DEGs. As pGGO is recognized as a component of TNM Stage IA LUAD according to the AJCC 8th edition TNM staging system, we evaluated the predictive ability of the 15-gene risk signature in patients with Stage IA LUAD. Patients with Stage IA LUAD and high-risk scores had worse OS than patients with Stage IA LUAD and low-risk scores, but the prognosis of high-risk patients with Stage IA LUAD was almost identical to that of patients with Stage II LUAD. These data suggest that treatment strategies for patients with Stage II LUAD may be beneficial in high-risk patients with Stage IA LUAD. The clinical application of the 15-gene risk signature was verified in two GEO datasets (GSE50081 and GSE72094). Findings showed no significant difference in OS between patients with Stage IA LUAD and high-risk scores and patients with Stage II LUAD. ROC curve analysis and DCA confirmed the risk signature’s ability to predict Stage I LUAD in the TCGA cohort. An enhanced nomogram was constructed to facilitate the clinical application of the risk signature. The predictive ability of the nomogram was verified in patients with Stage I LUAD in the TCGA cohort and GEO datasets. ROC curve analysis and DCA indicated that the nomogram combining clinicopathologic characteristics and the 15-gene risk signature had better predictive performance than the 15-gene risk signature alone.

GSEA was used to further explore the 15-gene risk signature. Cell cycle, DNA replication, Parkinson’s disease, pyrimidine metabolism, and ribosome pathways were activated in patients with high-risk scores, while allograft rejection, asthma, the intestinal immune network for IgA production, primary immunodeficiency, and systemic lupus erythematosus pathways were activated in patients with low-risk scores. High-risk patients had higher TMB than low-risk patients, and patients with high-risk scores and high TMB values had a poor prognosis. These data imply that the tumor immune microenvironment may be a prognostic factor in patients with LUAD and that patients with low-risk scores and CTLA4-positive and PD1-negative tumors may benefit from immunotherapy.

Some of the DEGs in the gene risk signature have been characterized. *DKK1* is a specific inhibitor of the canonical Wnt pathway (45). *DKK1* may reduce tumor cell migration and invasion by inhibiting the expression of β-catenin (46). The downregulation of *DKK1* may allow tumor cells to escape NK-cell-mediated cytotoxicity. *FAIM2* has been identified as an antiapoptotic protein that may protect cells from Fas-induced apoptosis. *FAIM2* may promote bone metastasis through the Wnt signaling pathway in patients with non-small-cell lung cancer (47, 48). *FGF5* is involved in many biological processes, including embryonic development, mitosis, and cell growth by regulating the cell cycle and VEGF pathway (49, 50). *PCP2* is a member of the R2B subfamily and is considered a tumor suppressor that influences the development of many cancers (51). *PCP2* can regulate the proliferation and differentiation of megakaryocyte cells (51). The data characterizing the other DEGs in the 15-gene risk signature are limited.

To the authors’ knowledge, the present study is the first to focus on the prognostic significance of pGGO-related DEGs in early-stage LUAD. As GGO is considered the appearance of early lung cancer and an important prognostic parameter in early-stage LUAD, our pGGO-related gene signature may contribute to patient classification, and have a clinical value in the diagnosis of patients with early-stage LUAD, and inform individualized treatment decisions. Patients with early-stage LUAD have a relatively good prognosis; however, the current staging system is imprecise for prognostic prediction. There is a need for novel prognostic signatures that identify high-risk patients with early-stage LUAD and guide clinical practice. Several reports have described robust gene risk signatures in LUAD, but only a few have focused on early-stage LUAD. Krzystanek et al. analyzed the gene expression from seven published LUAD cohorts and developed a 7-gene prognostic signature to enable better stratification and treatment of patients with Stage I LUAD. Wu et al. used public LUAD cohorts to establish a 21-immune-related gene prognostic signature for estimating OS in early-stage LUAD, recognizing the importance of the immune system in cancer initiation and progression. Peng et al. identified DE lncRNAs in individual cancer patients by comparing the disrupted ordering of expression levels of lncRNAs to stable normal ordering. They developed two lncRNAs’ (C1orf132 and TMPO-AS1) prognostic signatures for patients with Stage I–II LUAD who had not received adjuvant therapy.

In conclusion, we constructed a 15-pGGO-related DEG risk signature to predict the prognosis of early-stage LUAD. Risk scores were calculated using the regression coefficients for these pGGO-related DEGs. Patients with Stage IA LUAD and high-risk scores had poor prognoses with mortality approaching patients with Stage II LUAD. Therefore, treatment strategies for patients with Stage II LUAD may be beneficial in high-risk patients with Stage IA LUAD. A prospective randomized clinical trial is needed to confirm these findings.

## Data Availability Statement

The datasets presented in this study can be found in online repositories. The names of the repository/repositories and accession number(s) can be found in the article/**Supplementary Material**.

## Author Contributions

ZZ and WY conceived and designed the experiments, performed the experiments, analyzed the data, prepared figures and/or tables, authored or reviewed drafts of the paper, and approved the final draft. DL analyzed the data. BH, QC, WP, XP, GT, YL, YT, MP, FY, and XW conceived and designed the experiments, authored or reviewed drafts of the paper, and approved the final draft.

## Funding

This work was supported by the National Natural Science Foundation of China (81972195, FY; 82072594, YT), Fundamental Research Funds for the Central Universities of Central South University (2021zzts0385), the Hunan Provincial Key Area R&D Programmes (2019SK2253, FY, XW; 2020K53424, 2021SK2013), and the Scientific Research Program of Hunan Provincial Health Commission (Grant number: 20201047).

## Conflict of Interest

The authors declare that the research was conducted in the absence of any commercial or financial relationships that could be construed as a potential conflict of interest.

## Publisher’s Note

All claims expressed in this article are solely those of the authors and do not necessarily represent those of their affiliated organizations, or those of the publisher, the editors and the reviewers. Any product that may be evaluated in this article, or claim that may be made by its manufacturer, is not guaranteed or endorsed by the publisher.

## Supplementary Material

Supplementary Figure 1 RT-PCR.
