# Supplementary figures and images for: A Machine-Learning Approach to Developing a Predictive Signature Based on Transcriptome Profiling of Ground-Glass Opacities for Accurate Classification and Exploring the Immune Microenvironment of Early-Stage LUAD
Source: Front Immunol. 2022 May 26;13:872387. doi: 10.3389/fimmu.2022.872387 (PMC9178173; doi:10.3389/fimmu.2022.872387)

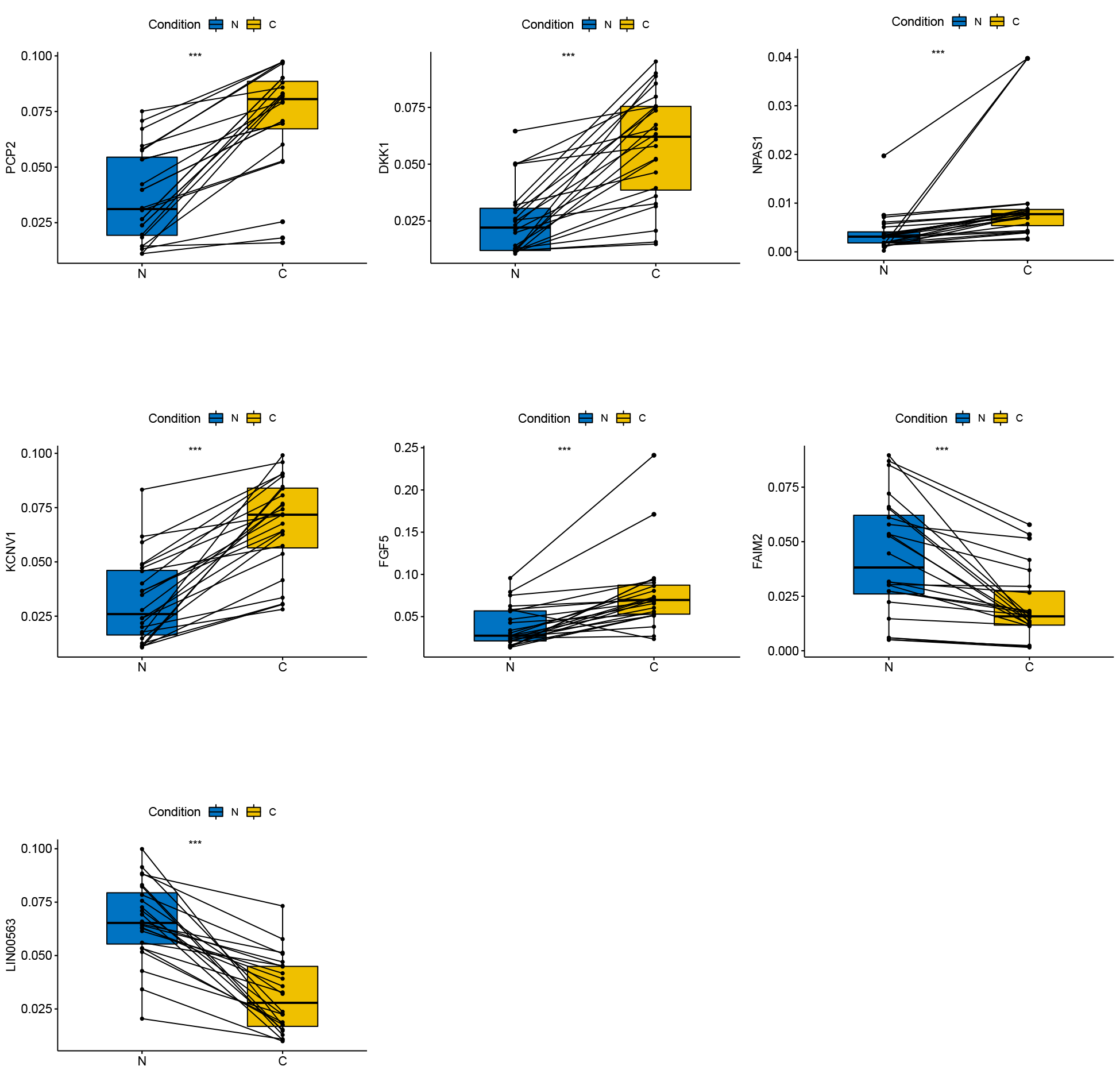

Supplement: Supplementary Figure 1 — RT-PCR. [file Image_1.tif]
